# Supplementary material for: Splice-Junction-Based Mapping of Alternative Isoforms in the Human Proteome
Source: Cell Rep. Author manuscript; Available in PMC 2020 Jan 15. (PMC6961840; doi:10.1016/j.celrep.2019.11.026)
Supplement: 2 [file NIHMS1546469-supplement-2.zip › DF1/PXD010154/Lung/Lung_2_PCBP2_YSTGSDSASFPHTTPSMCLNPDLEGPPLELTK.pdf]

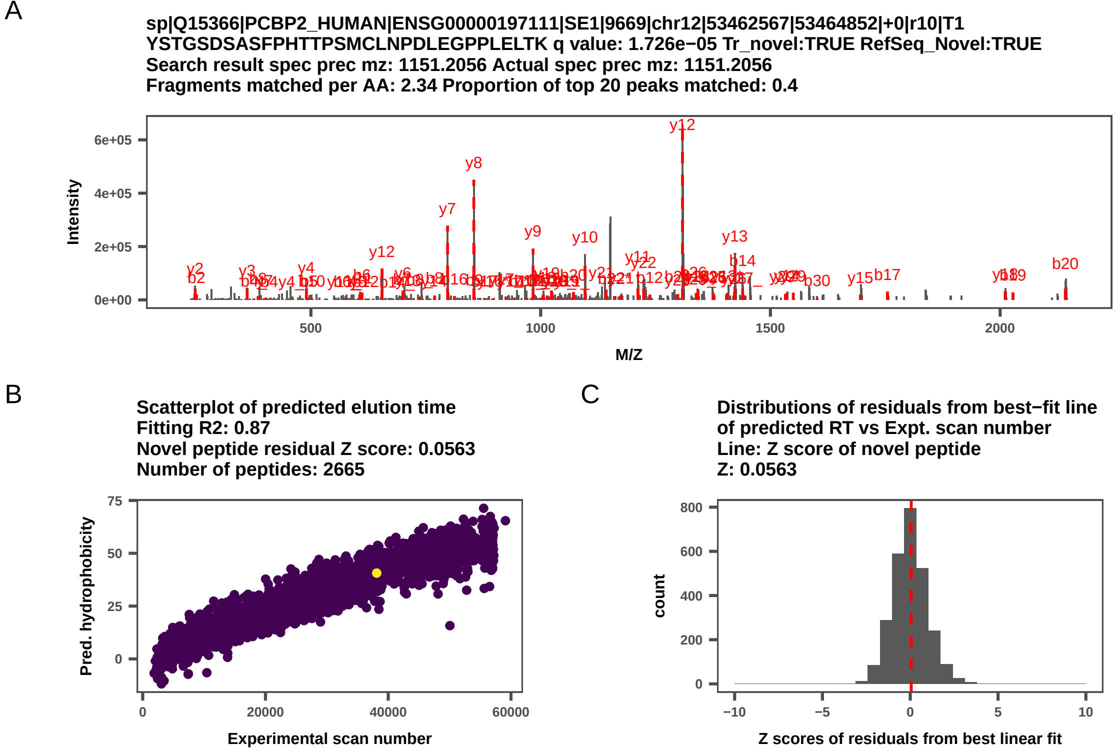
A
sp|Q15366|PCBP2_HUMAN|ENSG00000197111|SE1|9669|chr12|53462567|53464852|+0|r10|T1
YSTGSDSASFPHTTPSMCLNPDLEGPPLELTK q value: 1.726e−05 Tr_novel:TRUE RefSeq_Novel:TRUE
Search result spec prec mz: 1151.2056 Actual spec prec mz: 1151.2056
Fragments matched per AA: 2.34 Proportion of top 20 peaks matched: 0.4
Intensity
6e+05
4e+05
2e+05
0e+00
y12
y8
y7
y9
y10
y13
b14
y11
y22
y2
b2
y3
y4
y12
y6
y15
b17
b20
b30
500
1000
1500
2000
M/Z
B
Scatterplot of predicted elution time
Fitting R2: 0.87
Novel peptide residual Z score: 0.0563
Number of peptides: 2665
Pred. hydrophobicity
75
50
25
0
0
20000
40000
60000
Experimental scan number
C
Distributions of residuals from best−fit line
of predicted RT vs Expt. scan number
Line: Z score of novel peptide
Z: 0.0563
count
800
600
400
200
0
−10
−5
0
5
10
Z scores of residuals from best linear fit
